# Methods for multiple outcome meta-analysis of gene-expression data

**DOI:** 10.1016/j.mex.2020.100834

**Published:** 2020-02-21

**Authors:** Konstantina E. Vennou, Daniele Piovani, Panagiota I. Kontou, Stefanos Bonovas, Pantelis G. Bagos

**Affiliations:** a Department of Computer Science and Biomedical Informatics, University of Thessaly, Lamia 35131, Greece; b Department of Biomedical Sciences, Humanitas University, Milan, Italy; c IBD Center, Humanitas Clinical and Research Center - IRCCS, Milan, Italy

**Keywords:** Multiple outcome, Pleiotropic effects, Microarrays, Meta-analysis

## Abstract

Meta-analysis is a valuable tool for the synthesis of evidence across a wide range study types including high-throughput experiments such as genome-wide association studies (GWAS) and gene expression studies. There are situations though, in which we have multiple outcomes or multiple treatments, in which the multivariate meta-analysis framework which performs a joint modeling of the different quantities of interest may offer important advantages, such as increasing statistical power and allowing performing global tests. In this work we adapted the multivariate meta-analysis method and applied it in gene expression data. With this method we can test for pleiotropic effects, that is, for genes that influence both outcomes or discover genes that have a change in expression not detectable in the univariate method. We tested this method on data regarding inflammatory bowel disease (IBD), with its two main forms, Crohn’s disease (CD) and Ulcerative colitis (UC), sharing many clinical manifestations, but differing in the location and extent of inflammation and in complications. The Stata code is given in the Appendix and it is available at: www.compgen.org/tools/multivariate-microarrays.

• Multivariate meta-analysis method for gene expression data.
• Discover genes with pleiotropic effects.
• Differentially Expressed Genes (DEGs) identification in complex traits.

## Graphical abstract

**Figure:**
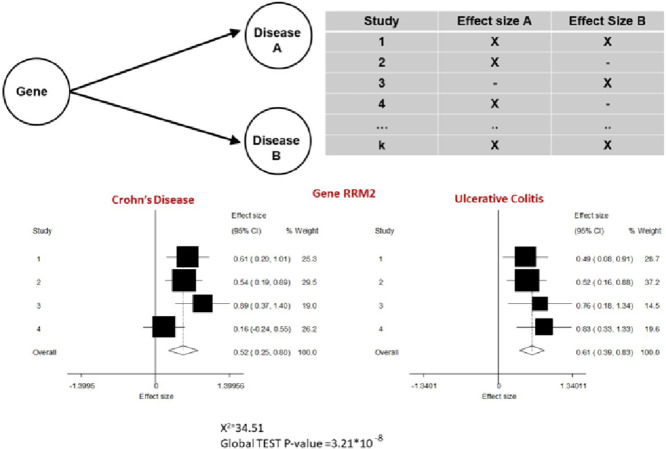

**Table:** Specification Table

Subject Area:	Biochemistry, Genetics and Molecular Biology
More specific subject area:	Bioinformatics
Method name:	Multivariate meta-analysis
Name and reference of original method:	This is a co-submission. The original method is: https://doi.org/10.1016/j.ygeno.2019.09.019
Resource availability:	The Stata code is available at www.compgen.org/tools/multivariate-microarrays and in supplementary material

## Method details

In gene expression experiments, the mRNA levels of thousands of genes are measured simultaneously in tissue samples. After the initial pre-processing steps that may differ among methods (normalization etc.), the identification of differentially expressed genes (DEGs^1^; genes that are differentially expressed, that is, up- or down-regulated, under different conditions) is a procedure usually reduced to a statistical test for the equality of means between groups (e.g. *t*-test). Concerning meta-analysis of microarray and RNAseq studies there are three broad categories of statistical methods that make use of effect sizes, p-values or ranks. For several reasons, the effect size approach which is based on common practices in meta-analysis, is preferable (in particular for the problem at hand) and thus it was advocated early in the literature [1]. However, to handle the problem of small sample size and non-normal data, most authors suggest a type of correction for calculating the statistical significance.

There are situations though, in which we have a comparison of more than two groups. In general, we may encounter multiple outcomes, multiple risk factors or multiple treatments. For instance, in a meta-analysis of microarray studies, there are cases in which different medications (treatments) are administered or different exposures (risk factors) are recorded. In another potential example several (related) diseases are investigated against a common healthy group (multiple outcomes). Such examples include diseases of similar etiology like autoimmune diseases (Multiple Sclerosis, Rheumatoid Arthritis and Type I Diabetes Mellitus), or diseases with similar manifestations, like Crohn's disease (CD^2^) and Ulcerative colitis (UC^3^). In all cases, we are usually interested in finding differentially expressed genes common in all conditions, or genes that differ among the conditions. However, the estimates obtained are not independent since a common comparison group is included, and the usual methods for univariate meta-analysis are not adequate, especially when it comes to comparing the estimates. The above-mentioned problems are dealt with in the framework of multivariate meta-analysis, in which we are interested in performing a joint modeling of the different quantities of interest [2], [3], [4]. The multivariate meta-analysis enables the researcher to perform global tests in order to avoid the inflation of type I error rate due to multiple comparisons. Additionally, one can estimate the covariance matrix for the parameters of interest, which could be of importance when one wants to perform a comparison for the equality of the two estimates and construct a confidence interval for the difference or some other function of the estimates [5]. Finally, a major advantage of multivariate meta-analysis is that it can accommodate, under the missing at random (MAR^4^) assumption, studies reporting only one of the parameters of interest, resulting in the so-called borrowing strength from external studies [6]. In all cases, applying a multivariate methodology and pooling data from multiple sources may prove important in increasing the power of the analysis and allowing, under a unified analysis, the identification of genes that play a central role in these conditions.

We used Stata^5^ [7], a general-purpose statistical software package widely used in biostatistics, epidemiology and econometrics. Stata allows user-written commands and there is an active community of scientists supporting the development of new methods. We implemented the multivariate meta-analysis method for gene expression data and we applied it to data regarding inflammatory bowel disease (IBD^6^), with its two main forms, CD and UC, sharing many clinical manifestations, but differing in the location and extent of inflammation and in complications. The application of the method and the results of the meta-analysis are discussed in detail in [8].

The model we use is based on the standard model of multivariate meta-analysis [2], [3], [4]. We are interested in comparing the mean difference of a continuous outcome (*X*), measured in study *i* = 1,2,…*k*. The effect sizes for the comparison of the two different disorders, are the standardized mean differences, which is referred to as Cohen's *d* [9]. It measures the difference in gene expression in CD and UC patients against controls (healthy subjects), respectively, and it will be estimated by:

(1) $${\hat{d}}_{1i}=\frac{{\bar{X}}_{1i}-{\bar{X}}_{0i}}{S_{\mathrm{pool}\mathrm{ed},i}}\;\text{and}\;{\hat{d}}_{2i}=\frac{{\bar{X}}_{2i}-{\bar{X}}_{0i}}{S_{\mathrm{pool}\mathrm{ed},i}}$$

Where *X*_1*i*_, *X*_2*i*_ and *X*_0*i*_ are the means of the expression of the particular gene in CD, UC and control groups respectively. *S_pooled,i_* is the pooled standard deviation, in study *i*, given by:

(2) $$S_{pooled,i}=\sqrt{\frac{\left(n_{0i}-\;1\right)S_{0i}^{2}+\;\left(n_{1i}-\;1\right)S_{1i}^{2}+\left(n_{2i}-\;1\right)S_{2i}^{2}}{n_{0i}+\;n_{1i}+\;n_{2i}-3}}$$

with *n*_1_*_i_, n*_2_*_i_* and *n*_0_*_i_* being the sample size of each group and *S*_1_*_i_, S*_2_*_i_* and *S*_0_*_i_* the gene expression standard deviation for each group.

The variance of the effect size estimates *d*_1_*_i_* and *d*_2_*_i_* will be estimated by:

(3) $$v\hat{a}r\left({\hat{d}}_{1i}\right)=s_{1i}^{2}=\frac{1}{n_{1i}}+\frac{1}{n_{0i}}+\frac{{{\hat{d}}_{1i}}^{2}}{2n_{i}},\;v\hat{a}r\left({\hat{d}}_{2i}\right)=s_{2i}^{2}=\frac{1}{n_{2i}}+\frac{1}{n_{0i}}+\frac{{{\hat{d}}_{2i}}^{2}}{2n_{i}}$$

and the covariance between the estimates of *d*_1_*_i_* and *d*_2_*_i_* will be

(4) $$c\hat{o}v\left({\hat{d}}_{1i},{\hat{d}}_{2i}\right)=\frac{\;1}{n_{0i}}+\;\frac{{\hat{d}}_{1i}{\hat{d}}_{2i}\;}{2n_{i}}$$

with *n_i_* = *n*_0_*_i_* + *n*_1_*_i_* + *n*_2_*_i_* being the total sample size of the study [10]. The calculation of the within-studies covariance is very important since it plays a crucial role in the multivariate method.

Several statistical methods have been proposed to handle the issues of small sample sizes efficiently and calculate accurate *p*-values and confidence intervals. These methods include resampling methods such as bootstrap and permutation, Bayesian methods and penalized or regularized *t*-tests. The bootstrap and permutation are easily applied but would require enormous computational time. Regularized *t*-tests on the other hand, require additional modifications for the multivariate setting. Here, we resort to an old but reliable alternative that has been shown to perform well, at least for moderate sample sizes (i.e. ~ 5–10 per condition). The idea is based on the fact that the sample estimates of the standardized mean difference *d* has the tendency to overestimate the absolute value in small samples. This bias introduced by *d* can be corrected using the so-called Hedges’ *g*, which generates an unbiased estimate. A correction factor, called *J*, is employed to convert from d to Hedges’ *g*.

(5) $${\hat{g}}_{1i}=J\left(\upsilon_{i}\right){\hat{d}}_{1i},\;{\hat{g}}_{2i}=J\left(\upsilon_{i}\right){\hat{d}}_{2i}$$

Although approximate formulae exist, the exact formula for *J* is given by [11]:

(6) $$J\left(\upsilon_{i}\right)=\frac{\Gamma \left(\frac{\upsilon_{i}}{2}\right)}{\sqrt{\frac{\upsilon_{i}}{2}}\Gamma \left(\frac{\upsilon_{i}-1}{2}\right)}$$

where *υ*_*ι*_ = *n*_0_*_i_*+ *n*_1_*_i_*+ *n*_2_*_i_* − 3. When *g* is used, the variances will also change to:

(7) $$v\hat{a}r\left({\hat{g}}_{1i}\right)={\left(J\left(\upsilon_{i}\right)\right)}^{2}v\hat{a}r\left({\hat{d}}_{1i}\right),\;v\hat{a}r\left({\hat{g}}_{2i}\right)={\left(J\left(\upsilon_{i}\right)\right)}^{2}v\hat{a}r\left({\hat{d}}_{2i}\right)$$

Similarly,

(8) $$c\hat{o}v\left({\hat{g}}_{1i},{\hat{g}}_{2i}\right)={\left(J\left(\upsilon_{i}\right)\right)}^{2}c\hat{o}v\left({\hat{d}}_{1i},{\hat{d}}_{2i}\right)$$

In the multivariate random-effects meta-analysis, we assume that ***g****_i_* = (*g*_1_*_i_, g*_2_*_i_*) is distributed following a multivariate normal distribution around the true means, according to the marginal model:

(9) $$g_{i}∼MVN\left(g,C+\Sigma_{i}\right)$$

By **Σ**_*i*_ we denote the within-studies covariance matrix:

(10) $$\Sigma_{i}=\left[\begin{array}{cc} s_{1i}^{2} & \rho_{W}s_{2i}s_{1i}\\ \rho_{W}s_{2i}s_{1i} & s_{2i}^{2} \end{array}\right]$$

The diagonal elements of **Σ**_*i*_ are the study-specific estimates of the variance (Eq. (3)), whereas the off-diagonal elements correspond to the pairwise within-studies covariances (Eq. (4)), for instance *cov*(*g*_1*i*_,*g*_2*i*_) = ρ_w_*s*_1*i*_*s*_2*i*_. It is important to note that the elements of **Σ**_*i*_ are considered known quantities. In contrast, by ***C*** we denote the between-studies covariance matrix, which is to be estimated during the fitting process:

(11) $$C=\left[\begin{array}{cc} \tau_{1}^{2} & \rho_{B}\tau_{1}\tau_{2}\\ \rho_{B}\tau_{1}\tau_{2} & \tau_{2}^{2} \end{array}\right]$$

A sometimes useful simplification can be made in Eq. (11) if we assume that the between-studies variances are equal [6]. In such case by letting *τ* = *τ*_1_ = τ_2_, ***C*** reduces to:

(12) $$C=\left[\begin{array}{cc} \tau^{2} & \rho \tau^{2}\\ \rho \tau^{2} & \tau^{2} \end{array}\right]$$

This is particularly useful in comparison of several treatments and in cases where the direct comparisons are scarce, since with this parameterization the total number of estimated parameters is reduced. In any case, the final marginal model on which we base the inference is:

(13) $$\left[\begin{array}{c} g_{1i}\\ g_{2i} \end{array}\right]∼MVN\left\{\left[\begin{array}{c} g_{1}\\ g_{2} \end{array}\right],\left[\begin{array}{cc} s_{1i}^{2}+\tau_{1}^{2} & \rho_{w}s_{1i}s_{2i}+\rho_{B}\tau_{1}\tau_{2}\\ \rho_{w}s_{1i}s_{2i}+\rho_{B}\tau_{1}\tau_{2} & s_{2i}^{2}+\tau_{2}^{2} \end{array}\right]\right\}$$

This model can be fitted in any statistical package capable of fitting random-effects weighted regression models with an arbitrary covariance matrix, such as SAS^7^, R^8^, or Stata. Stata code for fitting the model is presented in the Appendix using mvmeta which performs inferences based on either Maximum Likelihood (ML^9^), the Restricted Maximum Likelihood (REML^10^) or the multivariate method of moments (MM^11^), which is the method of choice due to its non-iterative nature [12].

As we already mentioned, a major advantage of this model is that it can accommodate, under the MAR assumption, studies reporting only one of the parameters of interest, resulting in borrowing strength from external studies [6]. Another advantage is that, with the estimated variance–covariance matrix, it can be used to perform global tests for the effect sizes. For instance, we can test the null hypothesis that both estimates of *g*_1_ and *g*_2_ are equal to zero (*H*_0_: *g*_1_ = 0, *g*_2_ = 0). The test statistic, *W*, follows a chi-square (*χ*^2^) distribution on 2 degrees of freedom and it can be obtained using the delta method:

(14) $$W={\left[\begin{array}{c} {\hat{g}}_{1}\\ {\hat{g}}_{2} \end{array}\right]}^{T}{\left[\begin{array}{cc} v\hat{a}r\left({\hat{g}}_{1}\right) & c\hat{o}v\left({\hat{g}}_{1},{\hat{g}}_{2}\right)\\ c\hat{o}v\left({\hat{g}}_{1},{\hat{g}}_{2}\right) & v\hat{a}r\left({\hat{g}}_{2}\right) \end{array}\right]}^{-1}\left[\begin{array}{c} {\hat{g}}_{1}\\ {\hat{g}}_{2} \end{array}\right]∼\chi_{2}^{2}$$

With this statistic one can test for pleiotropic effects, that is, for genes that influence both outcomes. More importantly, the test may discover genes that have a change in expression that is not detectable in the univariate setting. A similar test has been proposed for joint estimation of gene main effects and gene-environment interactions in GWAS^12^ [13] and by members of our team for identifying pleiotropic effects in bivariate meta-analysis of GWAS [14].

Another useful test, would be that of the equality of the estimates. In this case the null hypothesis (*H*_0_) would be: *D* = *g*_1_ − *g*_2_ = 0 and it can be tested using the Wald test (or an equivalent chi-square test):

(15) $$\frac{\hat{D}}{\sqrt{v\hat{a}r\left(\hat{D}\right)}}=\frac{{\hat{g}}_{1}-{\hat{g}}_{2}}{\sqrt{v\hat{a}r\left({\hat{g}}_{1}-{\hat{g}}_{2}\right)}}∼N\left(0,1\right)$$

The variance of *D*, would be obtained easily by the delta method:

(16) $$v\hat{a}r\left({\hat{g}}_{1}-{\hat{g}}_{2}\right)=v\hat{a}r\left({\hat{g}}_{1}\right)+v\hat{a}r\left({\hat{g}}_{2}\right)-2c\hat{o}v\left({\hat{g}}_{1},{\hat{g}}_{2}\right)$$

This test is similar to the test for the genetic model of inheritance in genetic association studies [15,16] and it can also, in principle, be used to perform a comparison of the two estimates and construct a confidence interval for the difference or some other function of the estimates.

As in every high-throughput method involving multiple testing, additional correction methods need to be applied afterwards to account for multiple-comparisons (multiple genes in this case) and various correction methods have been proposed for this task. These methods are grouped into two categories, the ones that control the family-wise error rate (FWER^13^) and the ones that control the False Discovery Rate (FDR^14^). The most common approach is to control the family-wise error rate using the Bonferroni [17] correction, which is easily applied and intuitive, but very conservative. Other popular methods used for multiple testing corrections are the methods proposed by Holm [18], Sidak [19] and Holland [20]. The most commonly used method in high-throughput experiments, however, is the method proposed by Benjamini and Hochberg, which instead of controlling FWER, controls the FDR [21]. FDR-controlling procedures are designed to control the expected proportion of rejected null hypotheses that were incorrect rejections. FDR-controlling procedures have greater power (i.e. they are able to detect more differences as statistically significant), at the cost of an increased Type I error rate. Stata implements easily most of the corrections mentioned above.

The method as presented here was applied to data regarding IBD, with its two main forms, CD and UC [8]. The datasets that were used in the multivariate meta-analysis contained data for 25,409 unique genes/loci from 251 CD patients, 175 UC patients and 159 healthy individuals, originating from 4 studies. The multivariate meta-analysis identified 249 statistically significant DEGs in patients with CD and 38 in patients with UC, at an FDR of 1%. The DEGs identified to be common to both diseases were 20. With the use of the global test (*W*) we identified 260 statistically significant DEGs that rejected the null hypothesis that both *g*_1_, and *g*_2_ are equal to zero. From the 260 statistically significantly DEGs, 53 were found to be statistically significant only by the global test and not in any of the diseases. These genes could not have been identified using univariate methods. The global test (*D*) was used as well, but only two genes (TRIP6 and IGHG1) were found to have equal change in the expression levels. Afterwards, we used these DEGs for further investigation with the help of bioinformatics overrepresentation analysis (ORA^15^) methods, that identified significant pathways [8].

The method was applied to microarray data; however, it can easily be applied also to RNAseq data. As we already mentioned, a major difference of Microarrays and RNAseq lies in the pre-processing steps, but for the purposes of a meta-analysis the analyst will not have to deal with that; one will assume that the data are processed and normalized. In general, data from microarrays and RNAseq are analyzed using the same meta-analysis methods [22,23], and in some cases, they are even combined together [24]. Taking these into account, we can safely assume that the method will work easily with RNAseq data, except for the case of a very small number of replicates (*n* = 2 or 3 per condition, which of course will be a problem also for microarrays). In this case, even though the correction method we used is reported to perform well, other more advanced options may be preferable (for instance to use a bootstrap correction).

To conclude, we have presented an application of the multivariate meta-analysis to gene expression data. The Stata code is given in the Appendix and it is available at www.compgen.org/tools/multivariate-microarrays. Contrary to common microarrays data formats, the code requires that samples are listed one per line, whereas the expression for the various genes (or probes) are given in columns. Moreover, numerical variables indicating the type of the sample (in the particular example: Healthy, UC, CD) and the actual study, are needed. It is the user's responsibility to ensure that the variables are coded appropriately. We need to mention that the method does not need all outcomes to be measured in every study. As we noted earlier, under the MAR assumption the method can incorporate studies that consider only *g*_1_, only*g*_2_, or both and in this case the increase in power is larger. However, for the proper estimation of between studies covariance, it is advised that a reasonable number of studies should report both outcomes. The code also works if a gene is present only in one study, but in this case the results are based only on the data provided by the particular study. Nevertheless, we also coded Eqs. (14) and (15) to report such results in order to prevent information loss. The code is provided as a .do file and in its current form reads a fixed-name .dta file and outputs the results in two separate files, one for genes for which meta-analysis was conducted and one for those that appear only in a single study. The user may change the file names accordingly. Moreover, the range of genes which will be analyzed are hardcoded, but this is easy to change. In its current form the code can handle two correlated outcomes but changing the inner loop in order to incorporate more outcomes is trivial. Extending the method to allow for more difficult settings, like network meta-analysis for mixed-treatment comparisons [25], will need additional modifications, especially when it comes to model specification.

## Funding

This work is part of Konstantina's Vennou thesis, which is co-financed by Greece and the European Union (European Social Fund- ESF) through the Operational Programme «Human Resources Development, Education and Lifelong Learning» in the context of the project “Strengthening Human Resources Research Potential via Doctorate Research” (MIS-5000432), implemented by the State Scholarships Foundation (IKY).

## Footnotes

1 DEGs - Differentially Expressed Genes.

2 CD - Crohn's Disease.

3 UC - Ulcerative Colitis.

4 MAR - Missing at random.

5 Stata - Statistical software package created by StataCorp.

6 IBD - Inflammatory Bowel Disease.

7 SAS - Statistical Analysis System, a statistical software developed by SAS Institute.

8 R - Programming language and free software environment supported by the R Foundation for Statistical Computing.

9 ML - Maximum Likelihood.

10 REML - Restricted Maximum Likelihood.

11 MM - Method of Moments.

12 GWAS - Genome-Wide Association Studies.

13 FWER - Family-Wise Error Rate.

14 FDR - False Discovery Rate.

15 ORA – Overrepresentation analysis.
